# Determinants of parents' reticence toward vaccination in urban areas in Benin (West Africa)

**DOI:** 10.1186/1472-698X-9-S1-S14

**Published:** 2009-10-14

**Authors:** Léonard Fourn, Slim Haddad, Pierre Fournier, Roméo Gansey

**Affiliations:** 1 National University of Benin, Cotonou, Benin; 2 Centre de recherche du Centre hospitalier de l'Université de Montréal, Unité de Santé Internationale, Montréal, QC, H2W 1V1, Canada; 3 NGO Research, ASSOBREC, Cotonou, Benin

## Abstract

**Background:**

Despite the efforts of health authorities, vaccination coverage of targeted child populations is still poor in many regions. Parents' reticence has been identified as one cause of this situation. However, there is little data to explain the phenomenon that could support decision-making.

**Objective:**

The objective of the study was to uncover the determinants of this reticence toward vaccination among the religious population of the cities of Parakou and Cotonou in Benin.

**Methods:**

This was an exploratory study using a qualitative survey of 12 pastors and 30 faithful from churches that are vaccination-reticent and a control group of the same number of faithful belonging to other churches, all Christian. Individual and group interviews were carried out in the local language using a pre-established and pre-tested guide. The data collected underwent discourse content analysis focused on specific themes.

**Results:**

Analysis of the data reveals an erroneous perception of child vaccination. Those who are reticent say vaccination goes against the will of God, that it is a poison from the "white witch doctor", and that those who vaccinate their children are committing a sin. Members of the control group argued against this, but without conviction. They adhere to the principle of obedience to authority, a biblical precept invoked when the vaccinators oblige them to vaccinate their children. Other factors were identified that could explain the reticence, such as the tactlessness of the vaccinators, parents' previous experiences and false rumours about vaccination.

**Conclusion:**

The reasons for reticence are mainly related to parents' beliefs in religious principles that are sometimes poorly understood. To limit the spread of this phenomenon, more detailed information and negotiation between the health authorities and the pastors of these churches are essential.

**Abstract in French:**

See the full article online for a translation of this abstract in French.

## Abstract in French

See Additional file 1 for a translation of the abstract to this article in French.

## Background

African countries have experienced high infant mortality rates due to many infectious diseases that are avoidable through immunization. However, over the past several years, the vaccination of targeted child populations has reduced the risk of these deaths, thus making this intervention the highest priority for health authorities. Gaining control over the problems of vaccine supply, cold chain, and economic issues recently identified in urban settings [1] has resulted in improved immunization coverage that is, however, still not entirely adequate. This disparity in the protection of children is often due to certain parents' refusal to vaccinate their children [2], seen mainly among children whose parents are members of imported sects or religions. Such refusals are evidence of the reticence to vaccination found in many localities in Benin, most often in the cities. In fact, while opposition to vaccination may not be new among minority populations, its extension to all routine vaccinations on the basis of religious arguments has become worrisome for the effectiveness of the Expanded Program on Immunization (EPI). While the literature mentions the example of the Muslim states of Nigeria where parents accused the polio vaccines provided by UNICEF of being contaminated with sterilizing chemicals [3], there is little literature on non-vaccinated children of other religions.

Any upsurge in reticence presents a constant risk of resurgence in controllable infectious diseases. The lack of data on the perceptions of parents in these religions results in inadequate support for decision-making among health authorities.

How is it that, in the same Christian religion, some sects accept the vaccination of children and others oppose it? Why is vaccination rejected when vaccines are offered at no cost to all targeted children?

The aim of this study is to document the factors that lead parents in urban settings to adopt this reticent behaviour toward all vaccinations, in order to help limit the growth of this phenomenon and reduce disparities in immunization coverage among children.

Vaccination is an important strategy in pursuing the Millennium Development Goal of reducing high infant mortality in developing countries [4], and is an effective weapon in preventing and reducing disparities in life expectancy by lowering the incidence of deadly childhood illnesses. With the support of development partners, many African countries have made vaccination a high priority in the fight against poverty and have succeeded in increasing the immunization coverage of targeted populations [5].

Nevertheless, despite the efforts of health authorities, there has been a resurgence of measles epidemics in areas with low immunization coverage. The persistence of this and other vaccine-preventable diseases has raised questions about the determinants of this poor immunization coverage [6-8]. A recent study (Fourn L, Gansey R, Djego J: Equité d'accès aux soins et immunisation au Bénin. Rapport de recherche. Unité Internationale UdM/CRDI. 2005 [unpublished]) on equity of access and immunization in Benin highlighted economically poor areas and some religious sects that reject vaccination. The freedom enjoyed by religious sects in Africa has resulted in a multitude of imported religions [9] outside of the classic religions (Catholic, Protestant, Muslim). Once installed in a country, these sects spread gradually into bordering countries with their principles, beliefs, and practices. Over the past six years, the number of such sects has grown, predominantly in cities. In Benin, 10 or more have been identified in Cotonou and Parakou, and in some of these, the practice of child vaccination is forbidden, while the others appear to tolerate routine vaccination.

Parents' refusal of vaccination is not new in the literature, but the bases for this behaviour vary from one country to another. In France, for example, parents' reticence is expressed as reservations and doubts about the efficacy of certain vaccines. Thus, the current debate about the hepatitis B vaccine is so ingrained in this reasoning that, despite reassuring results from many studies, parents' reticence persists [10-13]. In Canada, the phenomenon of reticence is primarily marked by some parents' lack of conviction about the expected benefits of certain vaccines [14].

Many authors have investigated the underlying causes of poor immunization coverage in Africa, and the main factors mentioned have to do with adverse effects post-immunization and dysfunctional vaccination services [1,15]. Parents' religious beliefs are also impediments to the acceptance of routine vaccinations, depriving targeted children of the benefits of these preventive services. Moreover, disparities in immunization coverage within the same area raise questions about the causes of this situation, especially in urban settings where everything is done to provide services at no charge.

The literature provides very few studies on reticence, and these have looked primarily at the side effects of vaccinations and the perception of the risk to the community presented by non-vaccinated children. This perception varies depending on whether parents are educated or not [16]. Also, the sources of information about vaccination play an important role in its acceptability among parents, who are very attentive to the sometimes negative rumours about vaccination. Because information directed at parents is not always consistent, some parents have erroneous information about vaccination [14,16]. This misinformation is often encountered in particular environments such as churches, neighbourhoods, and towns that are economically disadvantaged. The sustainability of immunization gains in most francophone African countries depends on controlling the emergence of this reticent behaviour as well as on the quality of services offered to children targeted by the vaccination program.

## Methods

### Study framework

Our survey was carried out in two cities: Parakou, in the north of Benin, close to Nigeria, and Cotonou, in the south. Parakou, the Department capital of Borgou, one of Benin's 12 departments, has a population of nearly 200,000 made up of many ethnic groups. There are approximately 27,000 children under the age of five, and the infant mortality rate is above 84%. Life expectancy at birth is 57.3 years. There is a variety of religious practices, chief among them Islam, followed by many Christian and other sects, of which some come from Ghana. The level of education is low, particularly among young women, mothers of the children targeted for vaccination. The dominant health issues are infectious and parasitic diseases, and the city has both public and private health establishments. Vaccination facilities and the organizing of outreach and fixed strategy sessions fall under the responsibility of the departmental authorities. Vaccination coverage is poor (55.9%), and the economic context is not encouraging.

Cotonou, a cosmopolitan city with a population of 800,000, is both Benin's economic capital and also the Department capital of Littoral. It has a high rate of educated females (63%). Life expectancy at birth is 60 years. The primarily business-oriented economic context is more advantageous to households that are relatively well-to-do than to the poor.

Besides the traditional religions (Catholic, Protestant, and Islamic), there are also many sects, some of which come from Ghana, Togo, and Central Africa. Membership in these sects can vary between 250 and 1,500 followers. The pastors of these sects are said to possess "charismatic healing" through prayer, and they recruit followers (whether educated or not) from various ethnicities. Some of these sects have a nearly military form of organization ruled by discipline and adherence to the pastor's words. Inspired by biblical scripture, some sects are reticent toward child vaccination, while others accept this measure for preventing infectious diseases.

Vaccination of targeted children in Cotonou is also free and is organized primarily using a fixed strategy, in both private and public health facilities.

### Design

We carried out a descriptive exploratory study using a qualitative survey among the pastors of the churches (sects) that are reticent toward vaccination and of parents of targeted children, as well as control groups of pastors and church members from non-reticent sects. The study targeted certain Christian sects based on their number of followers, their variety, and the ease with which they propagate into the villages. The recruitment process was similar for both types of Christian sects (reticent and non-reticent).

In Parakou, we selected two vaccination-reticent sects from among the five in the city; these were selected non-randomly for their large numbers of followers (300 to 1,000). The pastors of these churches were recruited after two discussion sessions in which we obtained their consent. The pastors and their assistants who had agreed to participate in the survey (one pastor and two assistants for each church) were retained in the sample. The same approach was carried out with the non-reticent Christian sects to select six leaders who agreed to participate.

In Cotonou, the pastors agreed to participate and signed the consent document without conditions. The recruitment was done in the same way, with the same number of pastors and assistants in the vaccination-reticent and non-reticent groups. Altogether, 24 religious leaders in Parakou (n = 12) and Cotonou (n = 12) participated in the survey.

Through these meetings, we were able to obtain their authorization to interview members of their churches, who were advised of the presence of the surveyors at the Sunday church services preceding data collection. At the end of the church service, surveyors retained one member out of every 10 present at the service to be participants. This recruitment was carried out on three successive Sundays in the churches of the selected sects. We recruited 30 parents of targeted children in each of the reticent and non-reticent sects; ultimately, only mothers of children between the ages of 12 and 23 months were selected. Each was given a written consent form to read and sign, with a guarantee of confidentiality, anonymity, and the freedom to accept or decline to participate.

The approach used in Cotonou was the same as in Parakou, for both the leaders and the church members. Many church members refused to participate, and we were only able to recruit 26 mothers of EPI targeted children in reticent churches. The control group of 30 non-reticent church members was also selected from the Christian churches whose followers vaccinate their children.

We conducted a descriptive, qualitative survey, carried out in each participant's home, away from the church premises. We used a single semi-structured, pre-established interview guide, for both religious leaders and church members, which included items about their perception and opinion of vaccination and about why this intervention might be considered unacceptable. We also assessed levels of education, where individuals were considered educated if they had completed four or five years of primary school and non-educated if they had received no formal education. The surveyors transcribed the interviewees' statements on paper during the course of the interviews.

The mothers who were interviewed individually also participated in focus groups to explore in greater depth certain points raised in the interviews.

Three focus groups were organized in Parakou with mothers of targeted children, two from the reticent sects and one from the non-reticent sect. The sessions were held at some distance from the other church members and the leaders, near the surrounding walls of the churches, with the authorization of the pastors. A team consisting of a facilitator (a sociologist experienced in group discussions), an observer, and an assistant carried out the sessions. Each was conducted in the local language and lasted about two hours, giving each mother time to share her thoughts. The group discussion guide included items on the perception of vaccination, opinions about reticence and the reasons underlying parents' reticence about vaccination.

In Cotonou, we conducted three focus groups under the same conditions as in Parakou with the interviewees from reticent sects. The non-reticent interviewees all refused to participate in the session, despite efforts by the team to persuade them.

In addition, in both Parakou and Cotonou, participants refused to have their statements recorded. We were required to take notes on paper; at the end of each session, the notes taken by the three session leaders were compared. Also, the pastors participated only in individual interviews carried out in their homes.

Content analysis was carried out after the statements from the focus groups and the individual responses were transcribed in French. We then compressed the data according to major themes emerging from the participants' statements to arrive at key messages.

## Results

### Sample characteristics

The sample of the reticent sects (churches) in Parakou consisted of a majority of men (16, or 53%); fewer than half of this faith group were educated (13, or 43%) and their average age was 30.4 ± 7.4 years. Most of the female faithful were non-educated. The dominant ethnicities were Lokpa and Fon. In Cotonou, the faithful we recruited were older (38.2 ± 10.8 years), and more than half (14, or 53%) were educated; 15 (57%) were men. The dominant ethnicities were Fon and Goun, spread across many sects.

The non-reticent faithful in Parakou were on average 34.1 (± 10.5) years old, and more than half were educated (20, or 67%). There was a variety of ethnicities, with none being dominant. In Cotonou, they were in the same age range of 31.4 ± 7.0 years, with 20 (77%) being educated. The majority (16, or 53%) were female (see Table 1).

**Table 1:** Sample distribution of church members by their socio-demographic characteristics.

	Reticent		Non-reticent	
		
	Parakou (n = 30)	Cotonou (n = 26)	Parakou (n = 30)	Cotonou (n = 30)
Demographic				
Average age	30.0 ± 7.4*	38.2 ± 10.8	34.1 ± 10.5	31.4 ± 7.0
Males	16 (53%)	15 (57%)	16 (52%)	14 (47%)
Education				
0 years	17 (57%)	14 (54%)	10 (33%)	7 (23%)
1-4 years	10 (33%)	11 (42%)	5 (17%)	3 (10%)
≥ 5 years	3 (10%)	1 (04%)	15 (50%)	20 (67%)
Ethnicity				
Lokpa	15 (50%)	1 (3%)	-	-
Fon	8 (27%)	14 (54%)	20 (66%)	22 (73%)
Goun	24 (80%)	9 (34%)	6 (20%)	8 (26%)
Others	4 (14%)	2 (8%)	4 (14%)	1 (4%)
Churches (religions)				
Apostolic (Batingue, Truth, etc.)	14 (47%)	9 (30%)	3 (10%)	1 (3%)
Evangelical	9 (30%)	10 (38%)	9 (30%)	4 (12%)
Celestial Christian	3 (10%)	1 (3%)	3 (10%)	8 (26%)
Renaissance d'homme (Union Revival Church)	4 (14%)	7 (27%)	4 (13%)	15 (50%)
Church of the Redeemed	-	-	10 (33%)	2 (7%)

* Average age with chi-square.

The ages of the pastors in the reticent sects of the two cities ranged on average between 45 and 48 years, while the non-reticent pastors were apparently younger (35.7 to 40 years). They were all male, and few of the reticent pastors had completed secondary school. In contrast, of the non-reticent pastors, 10 (83%) in Cotonou had completed secondary school. They were of various ethnicities and were fluent in the local languages, which they used for preaching. They generally lived not far from their churches (see Table 2).

**Table 2:** Sample distribution of church leaders by their socio-demographic characteristics.

	Reticent		Non-reticent	
		
	Parakou (n = 6)	Cotonou (n = 6)	Parakou (n = 6)	Cotonou (n = 6)
Average age	45 ± 7.5*	48 ± 9.4	40 ± 10.0	35.7 ± 2.0
Males	6 (100%)	6 (100%)	6 (100%)	5 (83%)
Educated**	2(33%)	3(50%)	4(67%)	5(83%)
Catchment population	500-1500	300-800	1000-1500	1600-3000

* Average age with chi-square.

** Individuals were considered educated if they had completed 4-5 years of primary school.

### Perceptions of the pastors of reticent churches

Interviews with the pastors in both cities revealed a variety of perceptions, with no unanimously shared common ground. According to them, their churches' principles are founded on the idea that "God, protector of humankind, looks after all His faithful," who require only prayer to protect and heal them in times of illness.

It is undoubtedly the belief in the miraculous power of prayer that motivates some parents not to vaccinate their children. The pastors say that vaccinations make healthy children sick, alluding to potential adverse reactions to vaccines. They assert that vaccination is "a tool of the Devil" that will impede children's protection by God. Our observation was that these pastors do not understand the benefits of immunization, and thus instil in their followers a false understanding of vaccination, which they consider to be "something evil, a satanic practice aimed at leading the faithful away from the right path."

The faithful perceive vaccinating children against their parents' will to be a violation of the rights of both children and parents. They deplore that some vaccinators use the police to force them to vaccinate their children, and say that the vaccinators should leave parents free to make their own choices, observing the tenets of their faith or biblical precepts. According to them, prayer is the only means of obtaining God's protection against illness.

Other leaders consider vaccines to be "poison" with which the vaccinators want to inoculate the children at any price; they see vaccinators as "distributors of poison and of sin." Church members who disobey instructions and have their children vaccinated provoke their pastor's anger and discontent. One pastor, in explaining this situation, said, "as soon as I find out this has happened, I punish these followers before the divine wrath comes down on them, because they are disobeying God."

Some pastors invoke biblical passages, whose content appears to be poorly understood, to justify their attitude toward health services, and especially vaccination. It is in this regard that the biblical passage from Isaiah, chapter 55, on the free blessings of God, is often cited.

### Perceptions of pastors who are non-reticent toward vaccination

The discourse of non-reticent pastors on the acceptance of vaccination is ambiguous, especially when they suggest that each person has his or her own opinion on the question and that they do not impose theirs on their followers. They say they have their own children vaccinated in spite of themselves and that the faithful are free to have their children vaccinated or not, in obedience to the health authorities. This obedience is based on the biblical principle that "the faithful must obey authority." Their discourse does not appear particularly to promote vaccination; these pastors wait until the authorities or vaccination officers force them to accept this preventive intervention. This ambiguous position is clearly illustrated in a statement made by one of the pastors: "I respect the position of members of my church who refuse vaccination. We must remember that faith is manifested differently in each of us, even if we are in the same church, such that individual members of my church can have different positions."

These different statements come primarily from leaders who are uneducated and those with three to four years of schooling. The discourse of educated pastors (four to five years of primary schooling) is often contrary to that of the non-educated pastors. They have a positive appreciation of the benefits of vaccination and encourage vaccinators to take care of the children of the faithful who accept. They do not impose their ideology on the faithful, but suggest that vaccination constitutes "a form of man's management of the world." They accept that when the risk of physical illness is imminent, the faithful can protect themselves with medical services.

### Religious perception of vaccination among the reticent

The perception of the reticent faithful is the same as that of their religious leaders. According to them, vaccination is against the will of God; vaccinating a child is like making a "deal with the Devil"; the act of vaccination is seen as "the work of the white witch doctor, contrary to biblical scriptures." The faithful of the churches in Cotonou declared without hesitation that vaccines are "poisons created by white people to harm us and to do experiments on us in giving us diseases."

Based on the principle that only God gives life and is responsible for protecting it against all diseases, the faithful are instructed to follow only God when faced with illness. "If we are with God, we must be only with God. We cannot mix things up: vaccination, herbal teas, talismans." They are convinced that vaccines make children sick. In one discussion group, a mother declared: "It is the vaccines that make our children sick: hot bodies, vomiting; I nearly lost my child because he was vaccinated by force the last time. His body became hot and he was vomiting."

### Personality of the pastor

In some churches, pastors have persuaded their followers that they can obtain cures for them from all sorts of illness, because they are healers with power from God. These pastors do not advise their followers to use health care services, and many followers believe wholeheartedly in the authority and healing power of their pastor.

From various conversations, it appears the aspirations and personalities of the religious leaders constitute real barriers to vaccination and promote reticence. In their quest for success and fame as healers, some pastors perceive health centres to be their competitors and therefore will portray them negatively, using primarily religious arguments. Some have constructed a taboo (a social and a moral proscription) around vaccination; in Benin, a taboo is often an object that must not be touched or an idea that must not be questioned. To defy this interdiction entails sanctions and reprisals from those who guard the taboo.

### Men and women, faced with a prohibition on vaccination

When men speak, they very often use a variety of citations to recall God's superiority. Women, on the other hand, refer to God as a superior being who must not be deceived and whose teachings must be respected. When the mothers of children targeted for vaccination spoke of God during the interviews, they often began with, "God does-n't like...", "God has forbidden...", "God said...", all of which may express their attachment to the directives of their religious leaders. Illustrations of this are: "God does-n't like things that are dirty, and your vaccines are dirty"; "God has ordered us to trust only Him. To use that which does not come from him to protect children is a sin."

### Perception of the faithful who are non-reticent toward vaccination

For the non-reticent, the advantages of vaccination were easily seen with the encouragement of vaccination officers. Nevertheless, concerns were raised about the forms of vaccines.

The group discussions and interviews carried out among the members of the churches in Parakou brought out the fact that vaccinations go under two names, depending on the form of administration. Vaccination by injection is called "Sopu" ("pricked"), while the oral form is called "Lisou". In Cotonou, the injectable form, "Abahwè", administered in the arm, is recognized and appreciated by mothers. They insist that no vaccine can be given "by mouth", whereas "Abahwè" has been known to them for generations. This may explain why oral vaccination is resisted by mothers in certain localities.

The faithful in other churches also declared that vaccination is "a means of prevention that can help save children." They acknowledge that it plays an important role as a barrier against deadly childhood diseases. For other mothers, an effective vaccination is recognizable by the indelible scar it leaves behind, which is not the case for many vaccinations in the EPI.

### Other identified deterrents to vaccination

In the localities of those who are reticent, church members with three to four years of schooling present the same arguments as those who are non-educated. In contrast, parents who completed primary school are more likely to be among the non-reticent.

Among all the deterrents mentioned, prior experience of vaccination seems to carry significant weight. Many mothers said that vaccination sessions involved bureaucratic hassles and resulted in expenses for travel and for medications, in cases of adverse effects.

False rumours about vaccination were mentioned to explain the reticent behaviour of some mothers, which undoubtedly arose from a lack of information. Thus, poorly informed mothers mistakenly attributed to vaccination the occurrence of anaemia, which is sometimes fatal. These are most certainly children with undetected anaemia who exhibit symptoms only after vaccination.

Other factors that incite some mothers to abstain from having their children vaccinated, even when they perceive the utility of it, include insufficient financial means to cover expenses and the family's fear of adverse reactions to the vaccination.

### Behaviour of health officials as a deterrent to vaccination

Some mothers who belong to reticent churches, even though they are reticent, have nevertheless had an experience of vaccination, which they recounted. They expressed their assessment of the two vaccination strategies generally employed and mentioned prior experiences that were negatively coloured by the tactless behaviours and actions of health workers. They said they preferred the outreach strategy, where the vaccinators are kinder and take their work more seriously, in contrast to vaccination sessions at fixed health posts. Negative behaviours on the part of vaccinators do not encourage mothers to use vaccination services.

## Discussion

Child vaccination remains an effective strategy against epidemics and a public health intervention with a good cost/benefit ratio [17]. However, coverage in some areas is still low, in spite of the efforts of vaccination teams. Recent studies have described the poor quality of vaccination services, with the constant stream of vaccine shortages, failures in asepsis with harmful consequences, and offensive behaviours on the part of vaccinators, all of which produce a negative reaction among parents in regard to immunization services in African countries [15,18]. In developed countries, the reticence observed arises from public dissatisfaction with information provided about vaccination and a desire to have more say in decisions. In France, for example, many parents express reservations and doubts about the efficacy of certain vaccines such as antigens against tuberculosis, measles, and mumps [16,19]. Their generally anti-establishment sentiments are furthered by the negative attitudes of some generalist physicians, a situation that encourages these parents in their rejection of vaccines of proven effectiveness. The situation was made worse by media reports on the risk of complications related to the measles vaccine and the risk of multiple sclerosis related to the hepatitis B vaccine [10,16]. Parents' reactions, denouncing these risks, raised doubts about the utility of vaccination.

The Nigerian study mentioned in the introduction to this paper described Muslim leaders' distrust of the poliomyelitis vaccine provided by UNICEF. They did not invoke Islamic belief in their refusal, but insisted the vaccine contained sterilizing products and accused the West of trying to rob them of their fertility [2,3]. In contrast, the present study focuses on religious belief among parents who belong to Christian sects as a factor in understanding the reticence toward vaccination.

Other studies have mentioned the religious dimension in explaining the low rate of immunization coverage, without going into detail [3,9,20]. However, the present survey of religious leaders and church members shows clearly the role played by religious factors in the occurrence and persistence of parents' vaccination-reticent behaviour. In their statements, parents insist on their belief, which is based on their own interpretation of biblical scripture. They see vaccination as the "white witch doctor's work" and not as a divine imperative. The term "witch doctor" designates a person with supernatural powers who has made a pact with the Devil. The white man has developed the vaccine, a product reputed to prevent diseases; yet only God has this power. By creating the vaccine, the white man has entered into competition with God and therefore must have made a pact with the Devil to acquire these supernatural powers.

Among the reticent pastors and the faithful, belief appears to be so strong that it has obliterated any difference between the faithful with up to three years of schooling and those who are non-educated (having never attended school). However, church-going parents and pastors who completed their primary education (four and more years) have a completely different behaviour; they acknowledge the benefits of vaccination for children's health and are generally not reticent.

The context within which the churches operate can also shed light on the decisions of the faithful with respect to vaccination. Indeed, the results of the survey show, among other things, that one difference between the non-reticent sects and those reticent toward vaccination is in the number of their members; the non-reticent sects have more followers than do those identified as vaccination-reticent. It may be that it is easier for a pastor to instil in a small number of followers his erroneous perception of vaccination and to impose this on them, than it would be with a larger number of people. Many parents of targeted children, and their pastors, demonstrated through their statements that their religious beliefs strongly prevented them from accepting vaccination against EPI diseases. Added to this is belief in the power of witchcraft practised by vaccinators and medical professionals, and especially by the white creators of vaccines. In developing a solution to reticence in the African and especially in the Beninois context, we must take into account the representation of vaccination agents as persons who compete with God by using vaccines as protection against disease.

In addition, from the statements of those in charge of the non-reticent churches and having low levels of schooling, we note that their perceptions of vaccination are not based on confidence. However, out of respect for an authority that orders vaccination and offers it free of charge to all parents, they accept to have their children vaccinated. This respect for authority was not shared by the reticent parents.

Besides this religious dimension of reticence, other factors were highlighted, related to the behaviours of vaccinators, the experiences of mothers, and false rumours. Most of these factors have already been mentioned in the literature from industrialized countries, where the preferred response has been to provide parents with clear information [19]. Indeed, in these industrialized countries, vaccination has been the victim of its own success. Because vaccination has defeated many infectious diseases, mothers of small children no longer live with the reality of these killer diseases and so no longer fear them, and they have difficulty comprehending the persistence of these diseases in other countries or localities [14,20]. In Africa, the situation is even more complicated because of the religious dimension that underlies reticence toward all child vaccination, and it requires a new response that takes into account the perceptions of parents in child immunization programs. It is important to reassure them with a broader base of information to counteract unfounded rumours and alleviate fears. From the results of this study, we observe a gap with respect to vaccination between the usual public health risk management model and unanticipated population behaviours. As suggested in the literature [14,19,21], a new form of dialogue is needed among all the actors involved in vaccination. Peaceful negotiation with reticent parents and their religious leaders, using resource persons from the same religion to vaccinate their children, would help to limit the expansion of this behaviour. Promotional actions should be aimed at correcting the poor understanding of religious scripture and communicating the benefits of this preventive intervention. These are essential elements of any negotiation with the leaders of the sects, whose power undermines the benefits that are sought from the vaccination programs in sub-Saharan Africa. Without such negotiation, there is a significant risk of losing the previous gains of the EPI at a time when efforts and resources currently directed at funding vaccines in the southern countries may become diverted toward dealing with the current food crisis.

Despite these results some limitations to the study were noted, mainly a non-randomized sample selection that reduced the generalizability of the results and no country data available on vaccination reticence. We note in passing that this study's results have galvanized many health authorities to embark on the national EPI team's pastor sensitization program to improve vaccination coverage.

## Conclusion

The reasons for reticence are mainly related to parents' beliefs in religious principles that are sometimes poorly understood. To limit the spread of this phenomenon, more detailed information and negotiation between the health authorities and the pastors of these churches are essential.

## Competing interests

The authors declare they have no competing interests.

## Authors' contributions

All authors participated in the interpretation of results and in the critical review of the paper.

## Supplementary Material

Additional file 1 Abstract in French. Click here for file
