# Supplementary material for: Determinants of parents' reticence toward vaccination in urban areas in Benin (West Africa)
Source: BMC Int Health Hum Rights. 2009 Oct 14;9(Suppl 1):S14. doi: 10.1186/1472-698X-9-S1-S14 (PMC3226233; doi:10.1186/1472-698X-9-S1-S14)
Supplement: Additional file 1 — Abstract in French. [file 1472-698X-9-S1-S14-S1.pdf]

Déterminants de la réticence des parents face à la vaccination dans les régions urbaines du Bénin (Afrique de l'Ouest).

Léonard Fourn, Slim Haddad, Pierre Fournier, Roméo Gansey

Résumé

Problématique

Malgré les efforts des autorités sanitaires, la couverture vaccinale des enfants cibles est encore faible dans plusieurs localités du Bénin. La réticence des parents à faire vacciner leurs enfants a été identifiée comme une des causes de cette situation. On ne dispose toutefois pas de bases factuelles permettant de mesurer l'ampleur et les effets du phénomène.

Objectif

L'objectif de l'étude était d'identifier les facteurs déterminants les comportements de réticence à la vaccination parmis des groupes religieux des deux principales concentrations urbaines du pays (Cotonou et Parakou).

Méthodes

L'étude repose sur une méthodologie qualitative. Des entrevues et des groupes focalisés de discussion ont été réalisés avec 12 pasteurs et 30 fidèles appartenant à des églises chrétiennes incitant les parents à ne pas vacciner leurs enfants. Un groupe témoin a été constitué d'un même effectif de fidèles appartenant à des églises n'étant pas défavorables à la vaccination. Les discussions ont eu lieu en langue locale après test des guides d'entrevue. Les analyses de contenu des discours ont été réalisées sur des thématiques précises.

Résultats

L'analyse des données révèle une perception erronée de la vaccination. Ceux qui s'opposent à la vaccination avancent que le vaccin est un acte non prescrit par Dieu ou transgressant les volontés divines. D'autres indiquent que c'est un poison du « sorcier blanc ». Ces justifications ne se retrouvent pas dans le groupe témoin, mais plusieurs semblent vacciner leurs enfants sans grande conviction, et cèdent aux infirmiers vaccinateurs au titre de leur respect de l'autorité. D'autres facteurs que religieux expliquent aussi la réticence : l'indélicatesse des vaccinateurs, l'expérience antérieure des parents, et la présence de fausses rumeurs concernant la vaccination

Conclusions

La réticence s'explique principalement par les croyances et les interdits religieux véhiculés par les églises locales. Pour enrayer ce phénomène, une meilleure information est requise et les autorités sanitaires doivent entrer en négociation avec les pasteurs des églises défavorables à la vaccination.
